# Tumeur à cellules de Sertoli-Leydig de l’ovaire: à propos d’un cas chez une jeune fille de 22 ans

**DOI:** 10.11604/pamj.2016.25.36.10469

**Published:** 2016-09-28

**Authors:** Diallo Moussa, Diouf Abdoul Aziz, Diallo Astou Coly Niassy, Koulimaya Cyre Espérence, Niang Youssou, Moreau Jean Charles, Diouf Alassane

**Affiliations:** 1 Centre Service de Gynécologie-Obstétrique, Centre Hospitalier National de Pikine Sis Camp de Thiaroye, Dakar, Sénégal; 2 Clinique Gynécologique et Obstétricale de l’Hôpital Aristide Le Dantec, Dakar, Sénégal

**Keywords:** Tumeur, Sertoli-leydig, ovaire, virilisation, Tumour, Sertoli-leydig, ovary, virilisation

## Abstract

Les tumeurs à cellules de Sertoli et Leydig sont des tumeurs sécrétantes rares du mésenchyme et des cordons sexuels. Cependant elles constituent l’une des tumeurs le souvent responsables de syndrome de virilisation. La certitude diagnostique est histologique après la chirurgie et il n’ y'a pas de signe échographie spécifique malgré la forte présomption clinique. Le pronostic comme la plupart des néoplasies est lié au degré de différenciation cellulaire et la présence d’éléments hétérologue en leur sein. L’objectif de notre travail était de rapporter un authentique syndrome de virilisation chez une jeune fille de 22 ans secondaire à une tumeur non épithéliale de l’ovaire à cellule de Sertoli et à cellule de Leydig. Les formes peu différenciées des tumeurs de Sertoli-Leydig ont un potentiel de malignité non négligeable. Le traitement est chirurgical, la chimiothérapie par association de sels de platine et de taxanes constitue un adjuvant intéressant. Le pronostic après la chirurgie est dominé par des récidives.

## Abstract (en)

Sertoli-Leydig cell tumors are rare secreting mesenchymal and sex cord-stromal tumors. However, they constitute one type of tumor most often responsible for virilization syndrome. A definite diagnosis is provided by histological examination following surgical excision of the tumor. It has no characterizing features on ultrasonography, in spite of the strong clinical presumption. Like many neoplasias, prognosis is related to the degree of cellular differentiation and to the presence of heterologous elements. The aim of our study was to report the case of a 22-year old woman suffering from a real virilization syndrome secondary to non-epithelial Sertoli-Leydig cell tumor of the ovary. Poorly differentiated Sertoli-Leydig tumors have high malignant potential. Treatment is surgical; taxane-platinum combination chemotherapy is an interesting adjuvant. Prognosis after surgical resection is related to the risk of relapses.

## Introduction

Les tumeurs de Sertoli-Leydig sont définies par l’OMS comme des tumeurs formées de cellules de Sertoli et de cellules de Leydig en proportions variables plus ou moins associées à un stroma primitif, et à des éléments hétérologues [1]. Elles sont très rares et représentent moins de 0,2% de l’ensemble des tumeurs de l’ovaire [2]. Elles font partie du groupe des tumeurs mésenchymateuses et des cordons sexuels. Elles surviennent fréquemment chez la femme jeune, entre 23 et 25 ans, cependant, il n’est pas rare de les observer chez les patientes de plus de 45 ans, voire après la ménopause [2, 3]. Elles constituent la principale tumeur féminine sécrétante responsable de virilisation. La prise en charge repose sur la chirurgie qui est très souvent mutilante à cet âge. Le pronostic est quant à lui lié au degré de différenciation cellulaire et de la présence d’éléments hétérologues. Nous rapportons ici l’observation d’une jeune fille de 22 ans qui a présenté une tumeur unilatérale de l’ovaire à cellules de Sertoli-Leydig peu différenciée.

## Patient et observation

Mlle F. F., nulligeste de 22 ans, avait consulté pour une augmentation du volume abdominal associée à une aménorrhée secondaire de 4 mois. L’examen clinique avait retrouvé une volumineuse masse ferme et sensible, à développement abdomino-pelvien, remontant au dessus de l’ombilic. Ailleurs, on notait un hirsutisme, une acné d’apparition récente et une pilosité pubienne de type masculin. L’échographie avait objectivé une masse occupant la quasi-totalité de l’abdomen mesurant plus de 25 cm de diamètre. L’échostructure était hétérogène, la composition mixte à prédominance liquidienne, bien limitée sans rapport avec les organes environnants. Le rein, le foie, la rate et le pancréas étaient normaux. La tomodensitométrie révélait un volumineux kyste organique de l’ovaire avec rehaussement périphérique cloisonnée sans signe de compression. Le CA 125 était à 17,17 UI/ml. La laparotomie avait mis en évidence une masse solide avec des plages kystiques, mesurant 25 x 18 cm, à surface lisse, sans végétation exokystique, avec des zones d’adhérences péri-capsulaires lâches avec l’épiploon. L’utérus, l’annexe controlatérale, le péritoine et l’épiploon étaient macroscopiquement normaux. Une annexectomie unilatérale était réalisée sans incident. Les conclusions du pathologiste étaient en faveur d’une tumeur de Sertoli-Leydig peu différenciée avec une composante rétiforme. A l’immuno-histochimie, on notait un immuno-marquage essentiellement des cellules de Leydigavec l’anti-inhibine, un marquage à l’anti CD99 et des cellules cubiques bordant les cavités avec la cytokératine AE1/AE3. Le suivi de la patiente était marqué par une régression de l’acné, une régularisation du cycle menstruel et un dosage du CA 125 à 13,15 UI/ml. L’échographie pelvienne à 3 mois ne montrait pas d’anomalie de l’ovaire controlatéral.

## Discussion

Les tumeurs à cellules de Sertoli et de Leydig représentent 25% des tumeurs endocrines de l’ovaire. Elles sont rares avec une prévalence de 0,2% de l’ensemble des cancers de l’ovaire [2]. Ce sont des tumeurs dérivées du mésenchyme et des cordons sexuels qui regroupent toutes les phases du développement embryonnaire du testicule, depuis l’aspect stromal diffus et cordonal indifférencié jusqu’au tube de sertoli bien différencié [1]. En fonction des proportions variables en éléments sertoliens et leydigiens, ces tumeurs sont classées en 3 groupes : les formes bénignes bien différenciées qui sont sécrétrices dans 60%, les formes à différenciation intermédiaire (cellules de sertoli immature) et les formes peu différenciées, sarcomatoïdes ou rétiniformes, groupe auquel appartient notre patiente [1]. L’atteinte bilatérale et synchrone des deux ovaires est rare [4]. Des formes familiales de ces types de tumeurs ont été décrites [5]. Sur le plan clinique, l’âge moyen de survenue se situe autour de 25 ans [2, 4] comme pour notre patiente. Les symptômes ne sont pas spécifiques. Il peut s’agir d’un syndrome tumoral, des douleurs pelviennes ou de troubles des règles à type d’aménorrhée secondaire. Les signes de virilisation tels que l’hirsutisme, la raucité de la voix, l’hypertrophie clitoridienne, la déféminisation de la silhouette et les modifications du comportement psycho-sexuel peuvent être retrouvés dans 30 à 50% des cas. En plus des signes classiques sus-cités, certaines patientes en période prépubertaire peuvent présenter des manifestations d’hyper-œstrogénie telle une pseudopubertéisosexuelle précoce ou des méno-métrorragies. L’explication trouvée à cette hyper-œstrogénie serait une transformation de l’androstérone produite par les cellules tumorales en oestradiol [4, 6, 7].

A l’échographie, les tumeurs à cellules de Sertoli-Leydig apparaissent sous forme de masses hétérogènes tissulaires vascularisées avec des zones solides; dans les formes à cellules de Sertoli pures, elles sont volontiers multiloculées, associées à des zones liquidiennes anéchogènes Leur taille est variable pouvant atteindre une vingtaine de centimètre [8]. Le bilan hormonal dépend de la symptomalogie. En présence de signe de virilisation, il convient de doser les principaux androgènes majeurs sécrétés chez la femme : testostérone, delta-4-androstènedione, déhidroépiandrostérone, sulfate de déhydroépiandrostérone. En général, seuls les androgènes d’origine ovarienne sont élevés. En présence d’une aménorrhée, le dosage des androgènes sera couplé à celui des hormones hypophysaires (FSH, LH, Prolactine). Il importe cependant de ne pas systématiser le bilan hormonal devant un syndrome tumoral isolé [4, 6, 7]. D’autres marqueurs ont été proposés par certains auteurs dans le cadre d’une surveillance biologique, il s’agit de l’alpha fœto-protéine, l’inhibine A et l’inhibine B [9]. L’examen histologique confirme le diagnostic et permet d’en définir le grade en fonction des proportions variables des éléments sertoliens et leydigiens et de leur degré de différenciation. Les tumeurs bien différenciées sont celles à cellules de Sertoli pures, qui prennent l’aspect du testicule prépubaire, et des cellules de Leydig qui se développent au niveau du hile et les tumeurs mixtes, constituées de cellules de Sertoli séparées par des amas de cellules de Leydig. Les tumeurs à différenciation intermédiaire sont constituées de tubes de Sertoli immatures, associés à un stroma de fait de cellules de Leydig, en proportion minoritaire. Les formes peu différenciées sont constituées de cellules fusiformes, d’aspect pseudo-sarcomateux, rétiniformes, évoquant le rete testis. A noter que des éléments hétérologues peuvent être retrouvés dans ces deux dernières formes (tissus osseux, cartilage, épithélium de type gastro-intestinal, hépatocytes) [4, 9, 10]. Le traitement repose essentiellement sur la chirurgie et tient compte des facteurs pronostiques que sont le volume tumoral, la différenciation tumorale, l’intégrité capsulaire et l’importance des mitoses. L’idéal est d’être le moins mutilant et le plus optimal. Une annexectomie ou une ovariectomie unilatérale est possible chez les femmes jeunes en âge de procréer et désireuses de grossesses. Si le traitement conservateur est envisagé, une stadification péritonéale devra être associée avec des biopsies sur les lésions suspectes. L’examen extemporané n’a pu être fait pour notre cas, mais il n’y avait pas de lésion péritonéale suspecte à l’exploration. En l’absence de tout désir de grossesse, et devant des formes peu différenciées, le traitement devra être radical avec réalisation d’une hystérectomie non conservatrice associée à une omentectomie[4]. Le curage ganglionnaire n’a pas encore de bénéfice prouvé. La chimiothérapie adjuvante au besoin est calquée sur celle des tumeurs germinales malignes qui ont un mauvais pronostic. Elle utilise la bléomycine, étoposide et cisplatine (BEP). Actuellement les taxanes ont démontré leur efficacité avec une toxicité moindre que le protocole BEP. L’association cisplatine et taxanes pourrait être intéressante dans l’avenir [4]. Quelques publications rapportent une radiosensibilité de ces tumeurs, mais au prix d’une toxicité bien supérieure à celle de la chimiothérapie [5]. Le suivi est clinique, biologique est radiologique surtout après traitement conservateur. L’observatoire des tumeurs rares préconise de faire un suivi tous les trois mois les deux premières années, un suivi clinique et biologique tous les six mois, et radiologique tous les ans de la troisième à la cinquième année, puis annuel.

## Conclusion

Les tumeurs non épithéliales de l’ovaire dérivées du mésenchyme et des cordons sexuels sont rares, celles à cellules de Sertoli-Leydig le sont les plus. Les signes de virilisation d’origine ovarienne en sont la particularité. Les formes peu différenciées ont un potentiel de malignité non négligeable. Le traitement est chirurgical, la chimiothérapie par association de sels de platine et de taxanes constitue un adjuvant intéressant. Le pronostic après la chirurgie est dominé par des récidives.
